# A 30-Year Long-Term Experience in Appendix Neuroendocrine Neoplasms—Granting a Positive Outcome

**DOI:** 10.3390/cancers12061357

**Published:** 2020-05-26

**Authors:** João Vinagre, Jorge Pinheiro, Olga Martinho, Rui Manuel Reis, John Preto, Paula Soares, José Manuel Lopes

**Affiliations:** 1 Instituto de Investigação e Inovação em Saúde (i3S), 4200-135 Porto, Portugal; jvinagre@ipatimup.pt (J.V.); psoares@ipatimup.pt (P.S.); 2 Instituto de Patologia e Imunologia Molecular da Universidade do Porto (Ipatimup), 4200-135 Porto, Portugal; 3 Faculdade de Medicina da Universidade do Porto (FMUP), 4200-139 Porto, Portugal; jorge.nature@gmail.com (J.P.); jrpreto@gmail.com (J.P.); 4 Departmento de Patologia e Oncologia, Centro Hospitalar e Universitário de São João, 4200-139 Porto, Portugal; 5 Life and Health Sciences Research Institute (ICVS), School of Medicine, University of Minho, Campus de Gualtar, 4710-057 Braga, Portugal; olgamartinho@med.uminho.pt (O.M.); rreis@med.uminho.pt (R.M.R.); 6 Biomaterials, Biodegradables and Biomimetics (3Bs-PT), Government Associate Laboratory, 4805-017 Guimarães, Portugal; 7 Molecular Oncology Research Center, Barretos Cancer Hospital, Barretos, São Paulo 14784 400, Brazil; 8 Department of Surgery, Centro Hospitalar e Universitário de São João, 4200-139 Porto, Portugal

**Keywords:** appendix neuroendocrine neoplasm, carcinoid, indolent, cell cycle

## Abstract

Neuroendocrine neoplasms (NENs) are the most common tumor of the appendix and have an excellent prognosis. Appendiceal tumors diagnosed between 1989 and 2019 were reviewed, and clinical data were collected from patient files. Part of the series was immuno-profiled for markers related to cell cycle proliferation and/or senescence-type, apoptotic, and metastatic potential. Appendix NENs were detected in 74 patients, with 0.47% of incidence per appendectomy. The median age of the patients was 21.5 years, with two age peaks of incidence at 17.0 and 55.2 years. The median tumors size was 5.8 mm, and most were smaller than 10 mm. Lymphovascular and perineural invasion, as well as necrosis, was associated with larger tumor size. G1 tumors composed 96.0% of the cohort. The presence of moderate/strong p16 and the absent/low Bcl-2 expression was frequently observed and associated with a smaller size. This study represents one of the largest cohorts and with a long follow-up. For tumors smaller than 10 mm appendicectomy was sufficient as a curative procedure, as revealed by the good outcome. This series presented a 100% disease-free survival. The indolent phenotype of appendix NENs is supported by the expression of markers that point towards a strong inhibition of cell replication and growth inhibition.

## 1. Introduction

Neuroendocrine neoplasms (NENs) are the most common tumors of the appendix, and the great majority of them are well-differentiated and have an excellent prognosis [1,2,3,4,5]. The NENs of the appendix are reported in the literature to have an incidence of 0.15–0.6 per 100,000 persons per year and to be slightly more frequent in female patients, with the highest incidence in the 5th decade of life [3,6,7,8]. Most of the appendix NENs are incidental findings in a post-appendectomy specimen, with an incidence of 3–5 per 1000 patients undergoing this surgical procedure [1,5,7,9,10]. Nevertheless, appendix NEN is presumed not to be a plausible cause of acute appendicitis, especially due to its more frequent location in the tip of the appendix (≈70%) [11,12]. Other locations include the mid-appendix (5–20%) and less frequently the base (<10%) [12]. A carcinoid syndrome associated with these NENs is rarely reported, and it is mainly associated with patients presenting metastatic disease. Metastatic disease is a rare event in patients with appendix NENs. Prognosis is usually excellent with a 5-year overall survival rate (5-YSR) close to 100%, within limited tumor stages [3,4,12]. Inclusion of all tumor stages does not confer such a favorable prognosis, with a 5-YSR of 70% to 85% [5]. The tumor stage is the main criterion to predict its behavior and subsequent therapeutic approach. Stratification of appendiceal NENs is performed according to size, location within the appendix, and the extent of invasion into the mesoappendix [5]. 

Goblet cell adenocarcinoma (GCA) is a distinct and less frequent neoplasm that can be found in the appendix, with an incidence of 0.01–0.05 per 100,000 persons per year [13]. The GCA corresponds to a different entity, a rare subtype of mixed neuroendocrine–non-neuroendocrine neoplasms (MiNEN) [14], with more aggressive behavior, with a reported 5-YSR range between 40% and 75%; at diagnosis, around 10% of GCA are already spread to the liver, peritoneum, and the ovaries [12,15]. By protein expression profiling, it has been demonstrated that these tumors have a high proliferation rate and deregulation of the cell cycle, with upregulation of cyclin D1 (cyclin-dependent kinase D1, CCND1), p21 (cyclin-dependent kinase inhibitor 1, CDKN1A) and downregulation of p16 (cyclin-dependent kinase inhibitor 2A, CDKN2A) [16].

This study aimed to present a single-center experience of appendiceal NENs diagnosed and treated for 30 years and to evaluate immunophenotypically markers that are associated with an indolent phenotype. For this purpose, we studied the expression of proteins that regulate cell cycle (p16, p21, and Cyclin D1), which can modulate apoptosis (Bcl-2, B-cell lymphoma 2) and that were reported to be associated to metastatic-potential (RKIP, Raf kinase inhibitor protein).

## 2. Results

The clinicopathological data from the 30 years are resumed in Table 1. During this period, 16,936 appendectomies were performed. Appendiceal NENs were detected in 74 patients, 27 (36.5%) males and 47 females (63.5%). Of these, 65 were diagnosed in appendectomy specimens suspicious for acute appendicitis, 8 in colectomies for other causes, and 1 in the context of adnexectomy for ovarian tumors. Of the 65 cases with performed appendicectomy, four did not present histological evidence of acute appendicitis; the cases with no evidence of appendicitis presented smaller size (1.00 ± 0.35 vs. 6.81 ± 0.69, *p* = 0.037, Table S1). While the great majority of neuroendocrine tumors in younger patients were diagnosed in the context of appendicectomy for acute appendicitis, the diagnosis of appendix NEN in colectomies for other causes was performed at a significantly higher age (25.61 ± 2.20 vs. 55.80 ± 6.76, *p* < 0.000), Table S1. The incidence of appendix NENs per appendicectomies was 0.38% (65 out of 16,936) and, when stratified by decades, was 0.16% (1989–1999) to 0.25% (2000–2009) and 0.40% (2009–2019), Figure S1B.

**Figure 1:**
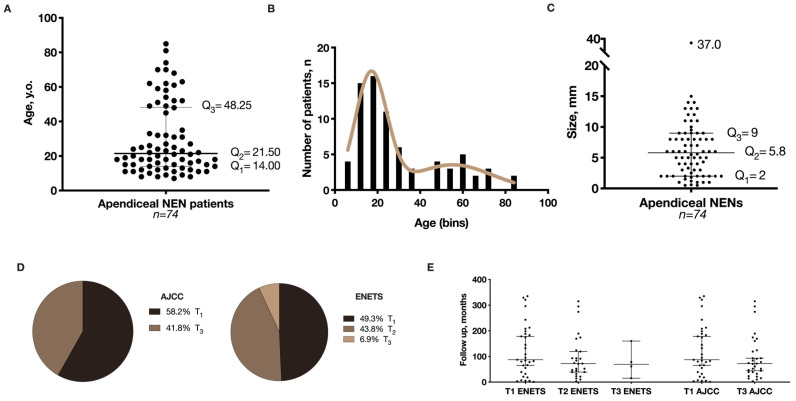
Graphical representation of (**A**) Age distribution of the appendiceal neuroendocrine neoplasms (NENs), median with interquartile range (IQR); (**B**) Bimodal distribution of age according to appendiceal NENs incidence; (**C**) Size distribution, median with IQR; (**D**) Patient staging (frequencies) according to European Neuroendocrine Tumour Society (ENETS) and the American Joint Committee on Cancer (AJCC) recommendations; and (**E**) Follow-up time distribution for ENETS and AJCC stage, median with IQR.

The median age at diagnosis was 21.5 years, Figure 1A, Table 1. Stratifying by age groups, young (<18 y.o., *n* = 26) and adults (≥18 y.o., *n* = 48), the median age was 12.0 and 31.5 y.o., respectively, Figure S1A. Non-linear fitting of the histogram representing age dispersion in 6-year bins revealed two peaks of higher incidence with mean ages of 17.0 and 55.2 years old, Figure 1B. The median size of the tumors was 5.8 mm (with a minimum tumor size of 0.5 mm and a maximum of 37 mm). The great majority of tumors were smaller than 20 mm (quartiles: Q_1_ = 2; Q_2_ = 5.8; Q_3_ = 9, mm), Figure 1C. Concerning location, most tumors were observed in the tip of the appendix 76.1% (*n* = 54), followed by the mid-appendix [18.3% (*n* = 13)], and less frequently in the base [5.6% (*n* = 4)], Table 1. Lymphovascular invasion, perineural invasion, and necrosis were identified in 12.3%, 16.4%, and 11.0% of tumors, respectively, and associated with larger tumor size, Table 1, and Table S1. Perineural invasion was observed more frequently in younger patients (15.00 ± 1.65 vs. 32.42 ± 2.78, *p* < 0.000), Table S1. Concerning grade, 96% were G1 (*n* = 70) and 4% were G2 (*n* = 3), Table 1; no G3 cases were identified. Concerning histological pattern, the insular pattern, not infrequently with prominent cytoplasmatic granules, was the most common (82.8%) and usually larger tumors (*p* < 0.039), Table S1. Trabecular and tubular patterns (features of L-cell type NENs) represented 17.2% of the cases, Table 1.

Regarding the depth of invasion, infiltration of the subserosa and mesoappendix was observed in 45.2% (*n* = 33), most <3mm, Table 1. As tumors infiltrated through the appendix layers, a stepwise increase in their size (*p* < 0.000) was observed, Table S1. Tumor–Node–Metastasis (TNM) staging system was performed according to the European Neuroendocrine Tumour Society (ENETS) and the American Joint Committee on Cancer (AJCC) guidelines [5,17]. According to ENETS, 36 patients with appendiceal NENs were staged as T_1_, 32 patients were staged as T_2_, and 5 as T3 stage. According to the AJCC staging system, 36 patients with appendiceal NENs were staged as T_1_ and 36 patients as T3 stage. Stage distribution and follow-up time points are represented in Figure 1D,E, and in detail in Table S2. Overall, patients presented a disease-free survival of 100%.

Lymph node staging was available in 11 cases. Only one case presented isolated tumor cells (ITC) in a single lymph node. Reviewing this specific case, it was a G2 tumor displaying the larger primary tumor size (37 mm) in our cohort. 

A follow-up scheme was offered to the patients based on the guidelines that were proposed/updated [5,18]. For the majority of the cases, due to the nature of the series (incidental neoplasms), curative resection of appendiceal NEN up to 1 cm by simple appendicectomy, and no specific follow-up strategy ensued in line with the update of guidelines [5,18] was assumed; the same applied to the cases with right-sided hemicolectomy without proof of lymph node involvement or any other residual disease. In the cohort nine patients presenting G1 appendiceal NEN with a size between 1 to 2 cm did not perform recommended right-side hemicolectomy; of these, two patients were lost, one died due to other causes, and the remaining six cases did not display risk factors (i.e., base location, mesoappendiceal invasion >3 mm, and angioinvasion) that would indicate them for specific follow-up due to postulated risk of lymph node metastases. The remarkable cases are the G2 NENs because two out of three patients did not receive the suitable right-side hemicolectomy. The logic behind this option was that both cases were diagnosed in 11-year-old patients where comorbidities of the surgical procedure were taken into account by the patients’ parents’ consciousness. To ease these circumstances, the patients are being followed by chromogranin A dosage and magnetic resonance imaging, as recommended [5,18]. One of these pediatric cases (with a 12 mm tumor, invasion of the mesoappendix (0.5 mm), mid-appendix location, and no pieces of evidence of lymphovascular invasion) was on follow-up for 14 months without evidence of disease. The other pediatric case (with a 14 mm tumor, a tip of the appendix location, minimal invasion of the mesoappendix (0.1 mm), lymphovascular and perineural invasion) was on follow-up for 174 months (14 years) without evidence of disease. The third G2 case was a patient subjected to right-hemicolectomy with a 37 mm tumor, with an invasion of the mesoappendix (3.5 mm) and other risk factors (lymphovascular and perineural invasion) already mentioned due to the presence of isolated tumor cells in a lymph node. At the end of the follow-up, no patient presented evidence of disease recurrence or died of the disease. 

**Figure 2:**
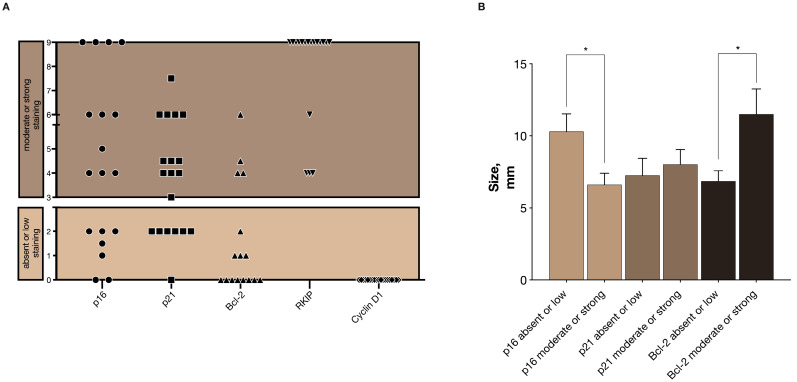
Representation of the final score estimation in the immunoexpression profiling: (**A**) Results of the immunoexpression profiling classified in absent or low staining and moderate or strong staining categories for p16, p21, Bcl-2, Raf kinase inhibitor protein (RKIP), and cyclin-dependent kinase D1 (Cyclin D1); and (**B**) Median size of the appendiceal NENs according to the molecular markers’ staining score, (bars with s.e.m., * *p* < 0.05).

For the evaluation of the immunohistochemistry markers, p16, p21, Bcl-2, RKIP, and Cyclin D1, we developed a semi-quantitative score based on the intensity and extension of the staining (Figure S1C). Representative images of the molecular markers’ expression are represented in Figure S2 and patient-related data in Table S3. The final score was grouped in absent/low expression or moderate/strong expressing categories. The presence of a moderate or strong p16 expression was observed in 61.1% of the appendix NENs (11 out of 18, Figure 2A) and associated with smaller tumor size (6.62 ± 0.78 vs. 10.29 ± 1.23, *p* = 0.017, Figure 2B), Table 2. For p21, 68.4% (13 out of 19) presented a moderate or strong expression, but no significant association with size was detected (Table 2). The anti-apoptotic protein Bcl-2 presented absent or low staining in the majority of the cases (75.0% (12 out of 16 cases)), and significantly associated with smaller tumor size (6.86 ± 0.72 vs. 11.50 ± 1.76, *p* = 0.011, Figure 2B), Table 2. All studied appendix NENs presented moderate and strong staining of RKIP. No expression of Cyclin D1 was detected for these tumors.

## 3. Discussion

The appendiceal NENs evaluated in this study represent one of the largest cohorts and run through a 30-year experience at Centro Hospitalar e Universitário de São João (CHUSJ). As in other series, most diagnosed appendiceal NENs had incidental findings in appendectomies specimens [3,5]. The incidence of the appendix NENs per appendectomy was 0.38% (3.8 per 1000 appendectomies), a value within the reported incidence of 3–5 per 1000 appendicectomies [5]. We detected an increase in incidence from 0.16% (1989–1999) to 0.25% (2000–2009), and 0.40% (2009–2019). This represents an increase of 156% (1989–2009), 160% (2000–2019), and 250% (1989–2019), in agreement with the data published by Singh et al. [19]; for the 1990s-decade we may have a partial underestimation due to non-computerized registry record. A slight preponderance for females was also observed in our cohort, in line with other reported series [3,5,8,12], and may be related to a higher incidence of appendicectomies performed in females [20]. The reported mean age at diagnosis ranges between 38 and 51 years [5]. We found a median age at diagnosis of 21.5 years, but expectable since CHUSJ is a reference center for pediatric surgery, performing a high number of appendectomies in children and young adults. We observed two peaks of incidence at 17.0 and 55.2 years old. In both age groups, most appendiceal NENs were diagnosed as an incidental finding in appendicectomy specimens for acute appendicitis. This corroborates that appendicitis is not an exclusive disease of younger people. Acute appendicitis ranks the second most common acute abdomen disease in patients over 50 years of age, conferring this bimodal incidence distribution with a maximum peak in adolescence and a second, smaller peak, in the elderly [21]. Most appendiceal NENs do not cause specific symptomatology. In our series, no case presented carcinoid syndrome [22], which is expected considering the small size and low stage of the tumors in the cohort. The presence of carcinoid syndrome in appendiceal NENs is rarely reported, usually associated with metastatic disease. Tumor location in the appendix of our cohort followed the reported series, being the tip the most common site [3,8,11]. 

No further treatment besides appendicectomy is recommended for tumors smaller than 10 mm. According to NCCN and ENETS protocols, right-hemicolectomy may be proposed if tumors are larger than 20 mm or in smaller if other risk factors are present [5,18]. The tumors in this cohort were mostly <10 mm, and due to its nature (incidental neoplasms), a curative resection with appendectomy not being subjected to follow-up was assumed; the same was applied to the cases with <10 mm that performed right-sided hemicolectomy. For the G1 appendiceal NENs with a size between 1 to 2 cm that did not obtain right-sided hemicolectomy, none presented evidence of risk factors (i.e., base location, mesoappendiceal invasion >3 mm, angioinvasion) that would select them to received regular follow-up due to presumed risk of lymph node metastases, in line with the ENETS guidelines. Still, we acknowledge that in this observational and retrospective study, despite no clinical evidence of recurrence, the follow-up may be considered not standardized to prove whether or not recurrence occurred. The noteworthy cases were the G2 cases, and two out of three patients did not receive the advisable right-side hemicolectomy due to their young age and are under a screening scheme as recommended [5,18]. The patients have been under follow-up for 14 and 174 months, and there was no evidence of disease. This is in line with the extent of the meta-analysis by Daskalkasis et al. [23], wherein pediatric patients had no strong morphological predictors for lymph node metastases. From the same study [23], and contrarily to pediatric cases, it was demonstrated that tumor larger than 20 mm, or more than 10 mm and/or with risk factors (lymphovascular perineural invasion) were associated with increased risk for lymph node metastases in adult patients. This was the case of our third G2 case, a patient subjected to right-hemicolectomy with a 37 mm tumor and multiple risk factors that presented isolated tumor cells in a lymph node; currently, the patient has 61 months of follow up with no evidence of disease. This case reflects the need for oncological radicalization when risk factors are present [24]. Notably, these three cases are a small snapshot of the current issues in appendix NENs where the true value of colectomy remains unsettled. Crown et al. [25] did not detect differences in recurrence-free and overall survival but confirmed the well-known comorbidities associated with prophylactic colectomy. Indeed, in our series with few cases of colectomy, complications were a cause of death, especially in older patients. In these 74 cases, none presented disease progression or recurrence. In fact, at the end of the follow-up, all patients were disease-free or died of other causes; the follow-up was quite long. This excellent survival rate may be in part related to the small size and low stage at diagnosis in the majority of tumors in our cohort, most being <10 mm, and it is reasonable to assume that appendicectomy may be considered curative in these cases. Considering this, our results are in line with the reported excellent prognosis of low stage appendiceal NENs, with 5-YSR close to 100% [3,4,5,12]. 

The indolent behavior of appendiceal NENs is well established in the literature [4,10,11,12], but there is a lack of studies addressing this behavior. Kanthan and colleagues [16] published an interesting study evaluating goblet cell adenocarcinoma (GCA) and reported that their high cellular proliferation rate was a consequence of cell cycle dysregulation, in particular, with upregulation of cyclin D1 and p21, and downregulation of p16. Since these proteins are closely related to senescence processes [26,27,28], we decided to evaluate them in appendiceal NENs, and complement the analysis with the evaluation of an anti-apoptotic protein Bcl-2. We also evaluated RKIP, a protein that has been associated with a metastasis suppressive role in various neoplasms, namely gastrointestinal stromal tumors, breast cancer, prostate, and many others [29,30,31,32,33]. Contrary to the GCA [16], in appendiceal NENs of our series, p16 was expressed in the majority of the cases with moderate or strong staining. This expression seems to damper a proliferative capacity of these tumors since, in comparison with the ones presenting absent or lower expression, they were significantly smaller. Besides other factors, the tumor size is also accountable due to the presence or absence of anti-apoptotic proteins; in particular, we observed that Bcl-2 expression was present in moderate or strong staining in tumors 2-times larger than in tumors with absent or low expression. In this study, p21 was not associated with clinicopathological features. Still, it was frequently expressed and may have a role in tumor suppression, contrasting with Cyclin D1 that did not seem to present a pivotal role as reported in GCA [16]. Due to the high expression of p16, it was expectable that Cyclin D1 would have a lower expression since the formation of the complex Cyclin D1 and CDK4 is inhibited by p16 [26,27,28]. Accordingly, we observed that Cyclin D1 expression was abolished. We also report that the high expression of RKIP in this appendiceal NENs is in line with extremely low metastatic rate commonly attributed to these NENs.

## 4. Materials and Methods 

### 4.1. Patients

The ethics committee of Centro Hospitalar e Universitário de São João approved the present study, and all procedures were following the institutional and national ethical rules (ethics code: 138/2007—tumores neuroendócrinos gastro-entero-pancreáticos). According to Portuguese law, informed consent is not required for retrospective studies. The 74 cases of appendiceal NEN diagnosed and treated at the Centro Hospitalar e Universitário de São João, Porto, Portugal, between March 1989 and March 2019 were retrospectively reviewed. Cases of goblet cell adenocarcinoma were excluded. Two experienced pathologists, J.P. and J.M.L., evaluated and reviewed the original specimens. Clinical data were collected from patient files, namely, patients’ age, gender, clinical presentation, surgical reports, and pathological reports referring to tumor size, location, and follow-up. TMN classification and grading of the appendiceal NENs collected were performed according to the European Neuroendocrine Tumour Society (ENETS) consensus guidelines and the American Joint Committee on Cancer (AJCC) guidelines for gastrointestinal neuroendocrine tumors [17,22]. Overall, follow-up data were retrieved from 65 patients with a mean follow-up period of 111.5 months and protocols from ENETS and National Comprehensive Cancer Network (NCCN) [18].

### 4.2. Immunohistochemistry

Representative 3-µm thick sections of the appendix were deparaffinized and rehydrated in a grading series of alcohols and water. Heat-induced epitope retrieval was performed in a decloaking chamber (Biocare Medical, Martinez, CA, USA) with the program, 1 minute at 98 °C and 2 min at 125 °C, for the antibodies p21, Cyclin D1, and Ki-67 in a 10 mM sodium citrate buffer at pH = 6. For the proteins p16 and Bcl-2, antigen retrieval was performed in a microwave set to 850 W until boiling and then 10 min at 150 W in an epitope retrieval solution pH = 9 (Leica Biosystems, Wetzlar, Germany). The immunohistochemical procedure was performed with the detection system Novocastra polymer detection system (Leica Biosystems) according to the manufacturer’s instructions. Primary antibodies were used with the following conditions: anti-p16^INK4a^ (551153, BD Biosciences, San Jose, CA, USA), 1:50, 1 h at room temperature (RT); anti-p21^WAF1^ clone EA10 (OP64, Merck Millipore, Burlington, MA, USA), 1:100, overnight at 4 °C; anti-Bcl-2 (NCL-L-bcl2, Leica Biosystems), 1:50, 1 h at RT; anti-Cyclin D1 clone SP4 (MA1-39546, Thermo Scientific, Waltham, MA, USA), 1:50, 30 min at RT; and anti-Ki-67 clone MIB-1 (M7240, Dako, Glostrup, Denmark), 1:100, 30 min at RT. For RKIP, a dilution of 1:1000 was used, and the previously reported conditions [31]. In all the experiments, positive and negative controls were used. The positive controls were breast cancer sample (p21 and Cyclin D1), normal tonsil sample (Bcl-2 and Ki-67), and human papilloma virus-infected cervical neoplastic tissue for p16. The negative controls were performed by omission of the primary antibody. Due to the optimal preservation of tissues and/or scarcity of material for the evaluation of all markers, only 19 of the 75 cases were evaluated. A final score for the p16, p21, Bcl-2, RKIP, and Cyclin D1 immunohistochemistry was generated by the multiplication of the intensity and extension values. The intensity of the immunoreaction was classified in absent = 0, low = 1, moderate = 2, and strong = 3. The extension score reflected the number of tumor cells presenting immunoreactivity: absent (0), 0 > *n* ≥ 25% (1), 25% > *n* ≥ 75% (2), 75% > *n* ≥ 100% (3). The final multiplicated score categorizes the samples in low or absent staining (scores 0 and <3, respectively) and moderate (scores ≥3 and <6) or strong staining (≥6) categories.

### 4.3. Statistical Analyses 

Statistical analyses were performed using IBM Corp. Released 2017, IBM SPSS Statistics for Mac, Version 25.0 (Armonk, NY, USA). The relationship between clinical–pathological parameters was evaluated by ANOVA and two-tailed unpaired Student’s *t*-test. The expression level (score) of the immunohistochemistry markers and clinicopathological parameters was evaluated by two-tailed unpaired Student’s *t*-test and Fisher’s exact test. Additional statistical and graphical analyses were performed using GraphPad Prism for Mac, Version 7.00 (La Jolla, CA, USA). A *p*-value < 0.05 was considered statistically significant.

## 5. Conclusions

In conclusion, we report a long-term series of appendiceal NENs in a reference center in Portugal. Our results are consistent with previous epidemiological data reports and also highlight a trend for the increased incidence of appendiceal NENs in the last decades. The molecular makers evaluated substantiated the expected better biological behavior of the appendiceal NENs. Indeed, they feature an indolent behavior that resembles a senescent-like phenotype. In particular, cell cycle markers expression (p16, p21, Cyclin D1, and Ki-67) might indicate that these tumors are putatively prone to a strong inhibition of cell replication (cell cycle arrest) as reflected by their low proliferative rate; tumor size hampering could also be accountable at the expenditure of anti-apoptotic protein expression. The absence of the expression of RKIP is in line with the clinical metastatic rate observed in the present series of appendiceal NENs. A lesson that we can take from this study is the peculiarity of the obtained results also reported in other studies: on the one hand, the point that well-differentiated appendix NENs in children and young adults can be cured with conservative procedures [23], and, on the other hand, from the same study and contrarily to pediatric cases, that tumors larger than 20 mm, or more than 10 mm and/or with risk factors (lymphovascular and perineural invasion) associated with increased risk for lymph node metastases in adult patients need to be addressed by hemicolectomy. Still, in the work by Crown et al. [25], recurrence-free and overall survival were equivalents regardless of surgical strategy. The latter allows us to question the cost/benefit when considering the comorbidities associated with the procedure. This duality we cannot truly clarify in the series we report, and it will remain disputable whether or not the common risk factors (size, lymphovascular/perineural, and mesoappendix invasion) are strong markers to indicate prophylactic hemicolectomy in this setting. All the same, our series is retrospective, carries limitations of follow-up procedures, and is enriched in lower grade and favorable stage cases, and does not hold power enough to project major findings, and prospective studies are needed to validate these eventual alterations.

## Figures and Tables

**Table 1:** Clinicopathological data of the appendiceal neuroendocrine neoplasms (NENs).

**Number of Appendectomies Performed**	**16,936**
**Number of Patients with Appendix NENs**	74
**Incidence of Appendix NENs in Appendectomies**	0.38%
**Gender**	
Male, n (%)	27 (36.5)
Female, n (%)	47 (63.5)
**Age at diagnosis (median), years**	21.5
<18, (median), years	12.0
≥18, (median), years	31.5
**Surgical procedure**	
Appendicectomy, n	62
Appendicectomy + right-sided hemicolectomy, n	3
Colectomy, n	8
Annexectomy, n	1
**Size**	
Median, mm	5.8
Location of the tumor *	
Tip of the appendix, n (%)	54 (76.1)
Mid-appendix, n (%)	13 (18.3)
Base of the appendix, n (%)	4 (5.6)
Histological pattern **	
Insular, n (%)	58 (82.8)
Trabecular / tubular, n (%)	12 (17.2)
Tumor infiltration ***	
Submucosa	16 (21.9)
Muscularis propria	24 (32.9)
Subserosa or mesoappendix	33 (45.2)
Lymphovascular invasion ***	
Yes	9 (12.3)
No	64 (87.7)
Perineural invasion ***	
Yes	12 (16.4)
No	61 (83.6)
Tumor necrosis ***	
Yes	8 (11.0)
No	65 (89.0)
Grading of the appendix NENs according to ENETS ***	
G1	70 (96.0)
G2	3 (4.0)
G3	0 (0.0)

* Three cases with data not assessed (na), ** Four cases with data na, *** One case with data na, European Neuroendocrine Tumour Society (ENETS).

**Table 2:** Molecular markers expression and tumor size association. Values in bold are statistically significant, *p* < 0.05.

Molecular Markers	Size (mean ± S.E.M.), mm	*p*-Value
p16 score (*n*)		
Absent or low (4)	10.29 ± 1.23	**0.017**
Moderate or strong (10)	6.62 ± 0.78	
p21 score (*n*)		
Absent or low (3)	7.25 ± 1.18	0.661
Moderate or strong (12)	8.02 ± 1.04	
Bcl-2 score (*n*)		
Absent or low (7)	6.86 ± 0.72	**0.011**
Moderate or strong (3)	11.50 ± 1.76	

## Supplementary Materials

The following are available online at https://www.mdpi.com/2072-6694/12/6/1357/s1, Figure S1: Stratification by age groups, incidence by decades and immunohistochemistry IHC semi-quantification, Figure S2: Representative images of the molecular markers p16, p21, Bcl-2, RKIP, Cyclin D1, and Ki-67 expression, Table S1: Clinicopathological associations, Table S2: Patients stage and follow-up data according to ENETS and AJCC, Table S3: Immunoprofiling of the markers p16, p21, Bcl-2, RKIP, and Cyclin D1 in appendiceal neoplasms.
