# Supplementary material for: A 30-Year Long-Term Experience in Appendix Neuroendocrine Neoplasms—Granting a Positive Outcome
Source: Cancers (Basel). 2020 May 26;12(6):1357. doi: 10.3390/cancers12061357 (PMC7353034; doi:10.3390/cancers12061357)
Supplement: Supplementary file 1 [file cancers-12-01357-s001.pdf]

A 30-year Long-Term Experience in Appendix Neuroendocrine Neoplasms—Granting a Positive Outcome

João Vinagre [1,2,3,4,†], Jorge Pinheiro [3,4,†], Olga Martinho [5,6,7], Rui Manuel Reis [5,6,7], John Preto [3,8], Paula Soares [1,2,3,4] and José Manuel Lopes [1,2,3,4,*]

Table S1. Clinicopathological associations.

	**Age** (years±S.E.M.)	***p*-value**
**Surgical procedure**		
Appendicectomy	25.61±2.20	***<0.000***
Other	55.80±6.76	
**Perineural invasion**		
Yes	15.00±1.65	***<0.000***
No	32.42±2.78	

	**Size** (mm±S.E.M.)	***p* value**
**Appendicectomy findings**		
Evidence of appendicitis	6.81±0.69	0.037
No evidence of appendicitis	1.00±0.35	
**Histological pattern**		
Insular	7.09±0.73	0.079
Trabecular / tubular	2.90±0.60	
**Lymphovascular invasion**		
Yes	11.67±0.49	0.001
No	5.65±3.26	
**Perineural invasion**		
Yes	11.37±0.49	0.000
No	5.36±2.48	
**Necrose**		
Yes	10.44±0.51	0.021
No	5.85±3.85	

	**Tumor infiltration**			***p*-value**
	**submucosa**	**muscularis**	**Subserosa or mesoappendix**	
**Size** (mean±S.E.M.), mm	3.00±0.65	4.58±0.68	9.36±1.03	<0.000

Table S2. Patients stage and follow-up data according to ENETS and AJCC.

Appendiceal NENs: Mean follow-up, (range), months	111.5 (1-335)

ENETS - Follow-up

	Number of patients	Follow-up median (range), months
$T_1N_0M_0$	4	80.0 (1-208)
$T_1N_xM_0$	32	129.4 (5-335)
**Total T_1**	**36**	**123.4 (1-335)**

	Number of patients	Follow-up median (range), months
$T_2N_0M_0$	7	112.8 (21-274)
$T_2N_xM_0$	25	93.1 (3-315)
**Total T_2**	**32**	**96.8 (3-315)**

	Number of patients	Follow-up median (range), months
$T_3N_0M_0$	2*	61.0 (-)
$T_3N_xM_0$	3	84.3 (15-160)
**Total T_3**	**5**	**78.5 (15-160)**

AJCC - Follow-up

	Number of patients	Follow-up median (range), months
$T_1N_0M_0$	4	80.0 (1-208)
$T_1N_xM_0$	33	129.4 (5-335)
**Total T_1**	**37**	**123.4 (1-335)**

	Number of patients	Follow-up median (range), months
$T_3N_0M_0$	8*	128.8 (52-274)
$T_3N_xM_0$	28	86.2 (3-315)
**Total T_3**	**36**	**94.4 (3-315)**

*1 case with isolated tumor cells in lymph node and one case without follow-up.

Table 3. Immunoprofiling of the markers p16, p21, Bcl-2, RKIP and Cyclin D1 in appendiceal neoplasms.

Age (y.o.)	Size (mm)	Grading	TNM (ENETS)	Status	Follow up (months)	p16			p21			Bcl-2			RKIP			Cyclin D1		
						Int	Ext	Score	Int	Ext	Score	Int	Ext	Score	Int	Ext	Score	Int	Ext	Score
45	4.3	G1	$T_2N_0M_0$	NED	274	3	3	9	3	2	6	0	0	0	3	3	9	0	0	0
32	8.0	G1	$T_1N_xM_0$	NED	132	3	3	9	1.5	3	4.5	1.5	3	4.5	3	3	9	0	0	0
81	8.0	G1	$T_2N_0M_0$	DOC	-	2	2	4	2	3	6	1	1	1	3	3	9	0	0	0
15	7.0	G1	$T_2N_xM_0$	NED	91	2	2	4	2	1	2	0	0	0	3	3	9	0	0	0
10	6.0	G1	$T_2N_xM_0$	NED	169	2	2	4	3	2	6	0	0	0	3	3	9	0	0	0
54	9.0	G1	$T_2N_0M_0$	DOC	93	2.5	2	5	2.5	3	7.5	1	1	1	3	3	9	0	0	0
62	8.0	G1	$T_1N_xM_0$	NED	84	3	3	9	2	1	2	1	1	1	3	3	9	0	0	0
22	6.0	G1	$T_2N_xM_0$	LOST	-	2	3	6	0	0	0	0	0	0	2	2	4	0	0	0
18	14.0	G1	$T_3N_xM_0$	NED	160	2	1	2	1.5	2	3	2	2	4	3	3	9	0	0	0
13	11.0	G1	$T_2N_xM_0$	NED	295	2	1	2	1	2	2	n.d.	n.d.	n.d.	2	2	4	0	0	0
58	9.0	G1	$T_2N_xM_0$	NED	119	2	1	2	1	2	2	2	3	6	3	3	9	0	0	0
31	3.0	G1	$T_1N_xM_0$	NED	329	n.d.	n.d.	n.d.	2	2	4	0	0	0	2	2	4	0	0	0
49	8.0	G1	$T_1N_0M_0$	NED	315	1	1	1	2	2	4	0	0	0	3	3	9	0	0	0
49	3.0	G1	$T_1N_xM_0$	NED	178	2	3	6	2	2	4	0	0	0	2	3	6	0	0	0
49	5.0	G1	$T_2N_0M_0$	NED	61	3	3	9	2	3	6	0	0	0	3	3	9	0	0	0
25	11.0	G1	$T_2N_xM_0$	DOC	47	0	0	0	1	2	2	2	2	4	3	3	9	0	0	0
21	15.0	G1	$T_2N_xM_0$	LOST	-	0	0	0	1.5	3	4.5	n.d.	n.d.	n.d.	3	3	9	0	0	0
14	2.5	G1	$T_1N_0M_0$	NED	66	2	3	6	1	2	2	n.d.	n.d.	n.d.	n.d.	n.d.	n.d.	0	0	0
63	9.0	G1	$T_3N_0M_0$	DOC	-	1.5	1	1.5	1.5	3	4.5	2	1	2	3	3	9	0	0	0

Intensity (Int): 3 – strong; 2 – moderate; 1 – low; and 0 – absence of expression. Extension (Ext): 3 – more than 75%; 2 – between 25 to 75%; 1 – less than 25%; and 0 – no cells stained. NED: No evidence of disease; DOC: dead of other causes; LOST: patient lost for follow-up. n.d.: Not determined due to tissue scarcity.

Figure S1

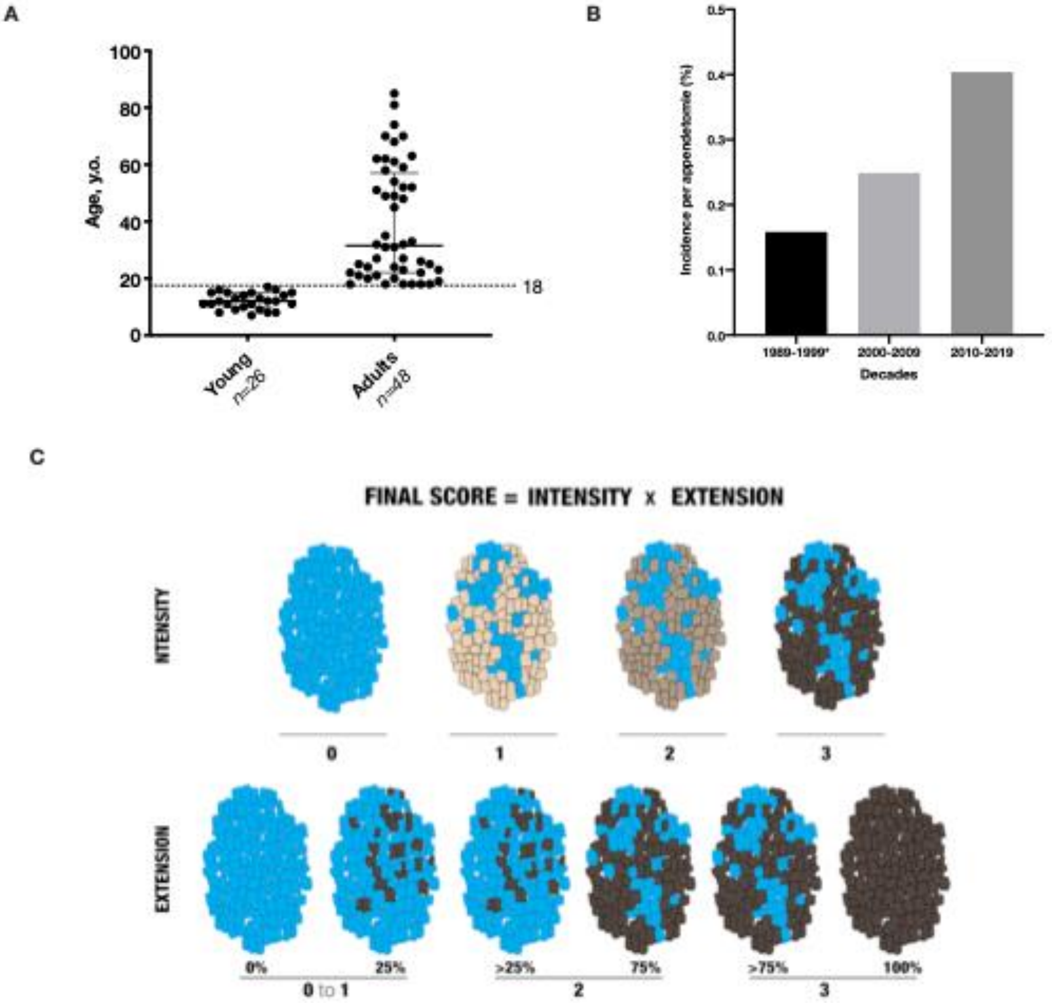

Figure S1. Stratification of the patients by age groups (A); the incidence of the appendiceal NENs by decades, * denotes that values from the '90s may be underestimated since during part of the decade the registry was not computerized; and IHC semi-quantification The intensity of the immunoreaction was classified in absent = 0, low = 1, moderate = 2 and strong = 3. The extension score reflected the number of tumour cells presenting immunoreactivity: absent (0), 0>n≥25% (1), 25%>n≥75% (2), 75%>n≥100% (3). The final multiplicated score categorizes the samples in low or absent staining (scores 0 and <3, respectively) and moderate (scores ≥3 and <6) or strong staining (≥6) categories.

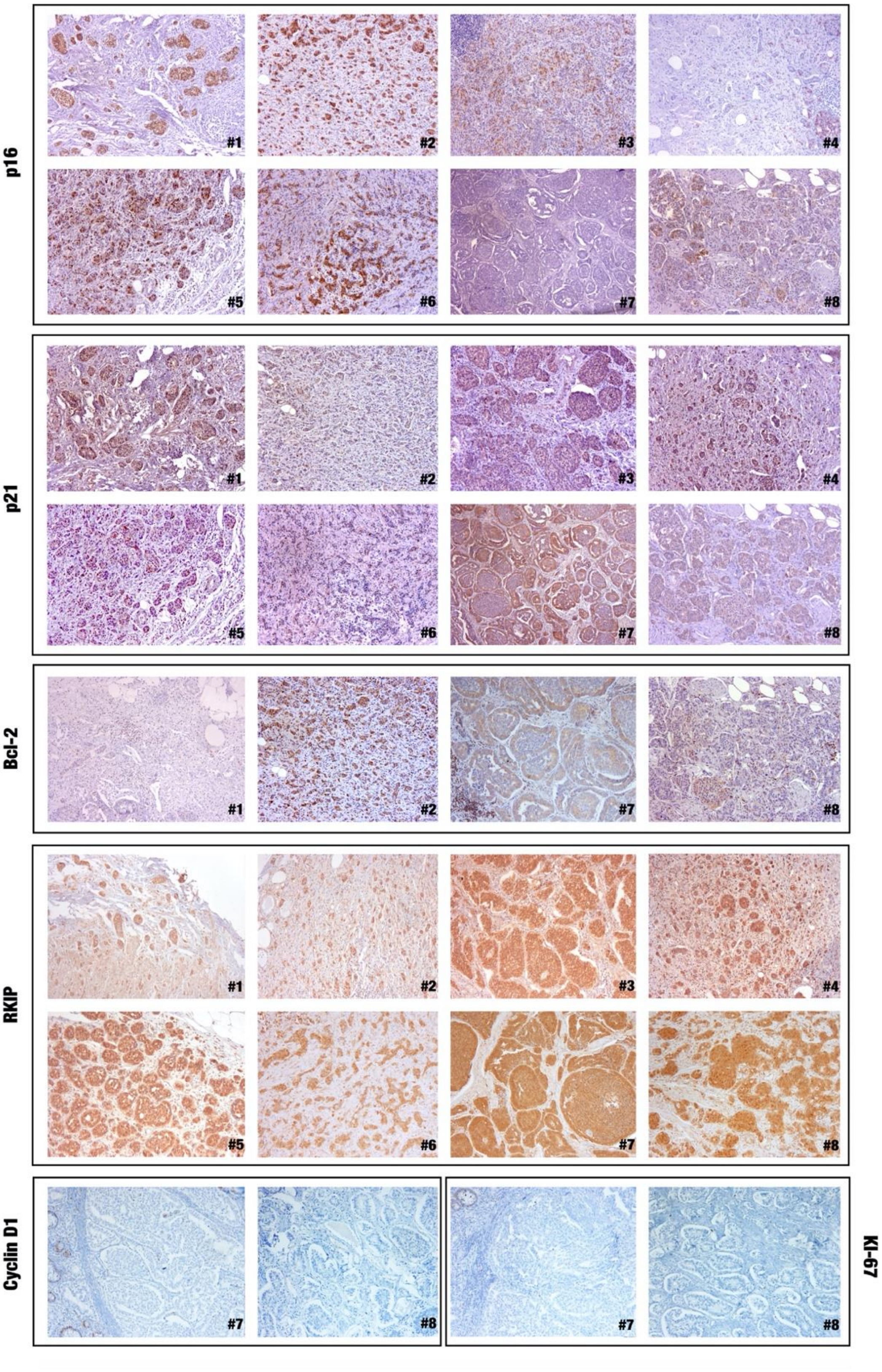

Figure S2 Representative images of the molecular markers p16, p21, Bcl-2, RKIP, Cyclin D1, and Ki-67 expression. #refers to the same case of appendiceal NEN with the different markers evaluated.
